# Improving the Completeness of Orthopaedic Operation Notes Through a Two-Cycle Clinical Audit at Prince Osman Digna Referral Hospital

**DOI:** 10.7759/cureus.102633

**Published:** 2026-01-30

**Authors:** Khaled Hassan Ibrahim, Hisham Eljack, Mohammed K Elbahi, Faris Jamalaldeen Mohammed Hamed, Mojtaba Mahmoud, Rawan AbdAlwahd Mohammed Osman Salih, Ahmed Abdelmonem Mahmoud, Ahmed Eltahir, Nada Eljaily Mohammed Babiker, Rayan Hashim Farah Mohammed, Almutassem Bellah Mojahed TajEldeen Ibrahim, Fatima S Mukhtar, Khalid A A Sallam, Adel Mahmah, Hagar A Mohamed, Shahd AlThaher, Sarah Elassad, Mazin S Khalil, Nada E Elnadi, Esraa A Elgendy, Aly A A. M. Morsy, Dina Elsharif, Ahmed Saiid Mohamud

**Affiliations:** 1 Orthopaedics and Traumatology, Bashair University Hospital, Khartoum, SDN; 2 Orthopaedics, Omdurman Military Hospital, Khartoum, SDN; 3 Surgery, Al Neelain University, Khartoum, SDN; 4 General Practice, Al Neelain University, Khartoum, SDN; 5 Orthopaedics, Prince Osman Digna Referral Hospital, Port Sudan, SDN; 6 Emergency Medicine, Port Sudan Teaching Hospital, Port Sudan, SDN; 7 Neurological Surgery, Al Neelain University, Khartoum, SDN; 8 Radiology, Hamad Medical Corporation, Doha, QAT; 9 General Practice, Bahçeşehir University, Istanbul, TUR; 10 Medicine and Surgery, Delta University for Science and Technology, Gamasa, EGY; 11 Medicine and Surgery, Jordan University of Science and Technology, Irbid, JOR; 12 Internal Medicine, Hamad Medical Corporation, Doha, QAT; 13 Medicine and Surgery, Georgian National University SEU, Tbilisi, GEO; 14 Medicine and Surgery, Kharkiv National Medical University, Kharkiv, UKR; 15 Medicine and Surgery, Mansoura University Hospitals, Mansoura, EGY; 16 Emergency Medicine, Alexandria University Main Hospital - Al Miri, Alexandria, EGY; 17 Medicine and Surgery, University of Kassala, Kassala, SDN; 18 Medicine and Surgery, Zamzam University of Science and Technology, Mogadishu, SOM

**Keywords:** clinical audit, low-resource settings, operation note documentation, orthopaedic surgery, patient safety, quality improvement

## Abstract

Background

Comprehensive operation notes are essential for patient safety, continuity of care, and medico-legal documentation, particularly in orthopaedic surgery where procedural complexity and the use of implants place greater demands on accurate operative documentation. Despite established guidelines, incomplete surgical documentation remains a common problem, particularly in resource-limited settings. This clinical audit aimed to assess and improve the completeness of orthopaedic operation note documentation at Prince Osman Digna Referral Hospital, Sudan.

Methods

A two-cycle clinical audit was conducted over a one-year period (2024-2025). In the first cycle, 50 orthopaedic operation notes were retrospectively evaluated against 18 criteria derived from the Royal College of Surgeons (RCS) Good Surgical Practice guidelines. Targeted interventions, including staff education, dissemination of standards, and senior-led supervision, were implemented. A second prospective audit of 35 operation notes was conducted three months later using the same criteria.

Results

Baseline compliance was poor for several critical documentation elements, with 0/50 (0%) of operation notes recording anticipated blood loss, antibiotic prophylaxis, and deep vein thrombosis (DVT) prophylaxis. Operative findings were documented in 17/50 (34%) of cases, while details of tissue altered were recorded in 16/50 (32%). Following the intervention, measurable improvements were observed across most domains; these changes represent meaningful relative gains compared with baseline documentation performance: anticipated blood loss documentation increased to 10/35 (28.6%), antibiotic prophylaxis to 7/35 (20%), and DVT prophylaxis to 5/35 (14.3%). Core procedural elements demonstrated high compliance in the second cycle, including documentation of the surgeon's name in 35/35 (100%), procedure name in 34/35 (97.1%), and postoperative instructions in 33/35 (94.3%).

Conclusion

This two-cycle clinical audit demonstrated measurable improvements in orthopaedic operation note completeness following targeted educational and supervisory interventions, particularly in descriptive operative elements such as the surgical approach, operative findings, and documentation of the tissue altered. However, documentation of key patient safety parameters, including anticipated blood loss, antibiotic prophylaxis, and DVT prophylaxis, remained suboptimal despite improvement from baseline. These findings indicate that while awareness-based interventions can improve documentation quality, the implementation of a standardized, mandatory operation note template is likely required to achieve consistent and sustained improvements in patient safety critical documentation.

## Introduction

The operative note represents the definitive record of a surgical procedure and serves as a cornerstone for postoperative management, multidisciplinary communication, clinical governance, and medico-legal accountability. High-quality surgical documentation is essential to ensure continuity of care, facilitate accurate handovers, and support patient safety initiatives. Recognizing its importance, the Royal College of Surgeons (RCS) emphasizes that operation notes should be clear, comprehensive, and completed contemporaneously as part of good surgical practice [1].

Despite established guidelines, incomplete and inconsistent operation note documentation remains a widespread problem across surgical specialties and healthcare systems. Previous audits have demonstrated that omissions in operative details, particularly regarding intraoperative findings, prophylactic measures, and postoperative plans, may lead to communication failures, delayed clinical decisions, and increased medico-legal risk [2,3]. These challenges are often more pronounced in resource-limited settings, where high patient volumes, time constraints, and a lack of standardized documentation tools may further compromise documentation quality.

Evidence suggests that structured quality improvement interventions, including clinical audit, feedback, education, and the use of standardized templates, can significantly improve the completeness of surgical documentation. Jedwab et al. reported improved compliance with documentation standards following targeted interventions, while similar audit-based initiatives in various surgical disciplines have shown sustained improvements when documentation standards are actively reinforced [2-4].

At Prince Osman Digna Referral Hospital, Sudan, informal departmental observations suggested considerable variability in the quality and completeness of orthopaedic operation notes. However, no systematic evaluation had previously been undertaken to quantify these deficiencies or assess compliance with international standards. This clinical audit was therefore designed to evaluate orthopaedic operation note documentation against RCS guidelines and to implement targeted interventions aimed at improving documentation quality through a structured audit cycle.

## Materials and methods

Study design and setting

This study was conducted as a clinical audit and quality improvement project within the Orthopaedic Department of Prince Osman Digna Referral Hospital, a tertiary referral center in Sudan. The audit followed a two-cycle design consistent with established quality improvement methodology and was carried out over a one-year period from May 2024 to May 2025.

Standards and audit criteria

Audit standards were derived from the RCS Good Surgical Practice guidelines, which outline essential elements required for comprehensive and safe operation note documentation [1]. Eighteen core documentation criteria were selected as predefined audit standards, encompassing patient and procedural identifiers, intraoperative details, and safety-related parameters. Two additional documentation items (postoperative instructions and surgeon signature) were recorded and reported separately for completeness but were not counted as part of the core audit criteria. Items considered "if applicable", such as prosthesis details, were assessed only in procedures where they were clinically relevant; in cases where these items were not applicable, they were recorded accordingly and were not treated as missing data.

First audit cycle

The first audit cycle was conducted retrospectively. Fifty consecutive orthopaedic operation notes were reviewed and assessed for compliance with the 18 predefined criteria. The audit included both elective and trauma cases performed in the main orthopaedic operating theatres. All legible handwritten operation notes were included, while notes that were severely illegible or incomplete to the extent that assessment was not possible were excluded. Data were collected using a structured checklist developed directly from RCS standards. Data extraction was performed by the audit team using a standardized data collection form, with random cross-checking of a subset of records to ensure consistency. All operation notes reviewed in the first audit cycle were handwritten, as electronic operation note documentation was not in use at the study center during the audit period. Each criterion was recorded as either documented or not documented.

Intervention

Based on the deficiencies identified during the first audit cycle, a multifaceted intervention was implemented. This included the following: formal presentation of audit findings during a departmental clinical governance meeting to raise awareness of documentation gaps; distribution and discussion of RCS operation note standards among orthopaedic surgical staff; direct feedback and reinforcement by senior surgeons during operating theatre sessions; and emphasis on the patient safety and medico-legal importance of complete operation note documentation.

Second audit cycle

Following a three-month intervention period, a second audit cycle was conducted prospectively. Thirty-five orthopaedic operation notes were reviewed using the same 18-point checklist to ensure consistency and comparability with the first cycle. Consecutive orthopaedic operation notes referred to all eligible notes recorded sequentially during the defined audit period, including both elective and trauma cases performed in the main orthopaedic operating theatres. All legible handwritten operation notes were included, while severely illegible notes were excluded.

Data analysis

Compliance with each documentation criterion was calculated as a percentage for both audit cycles. Results from the first and second cycles were compared descriptively to assess the impact of the implemented interventions. Given the audit-based nature of the project and sample size, formal inferential statistical testing was not performed.

Ethical considerations

This project was reviewed and approved by the Institutional Review Board of Prince Osman Digna Referral Hospital as a quality improvement project. Informed consent was waived as the audit involved retrospective and prospective review of clinical documentation only, with no patient identifiers collected or reported.

## Results

A total of 85 orthopaedic operation notes were reviewed across two audit cycles. The first audit cycle included 50 operation notes, while the second cycle evaluated 35 notes following the implementation of the targeted interventions.

First audit cycle

The baseline audit revealed significant deficiencies in the completeness of orthopaedic operation note documentation. While core identifiers were generally well recorded, such as the date of operation in 47/50 (94%), the name of the operating surgeon in 49/50 (98%), and the name of the operation in 48/50 (96%), several critical clinical and safety-related parameters were poorly documented.

Notably, none of the operation notes included documentation of anticipated blood loss 0/50 (0%), antibiotic prophylaxis 0/50 (0%), or deep vein thrombosis (DVT) prophylaxis 0/50 (0%). Descriptive operative details were also frequently omitted, including operative findings documented in 17/50 (34%), details of tissue removed or altered in 16/50 (32%), and the surgical approach in 20/50 (40%). Documentation of postoperative instructions was comparatively better, being present in 44/50 (88%) of operation notes.

For the purposes of this audit, documentation was considered compliant if the relevant parameter was explicitly mentioned in the operation note. Anticipated blood loss was considered documented if any numeric or narrative estimate was recorded. Antibiotic prophylaxis and DVT prophylaxis were considered documented if any explicit reference to their use was present, regardless of agent, dose, timing, or modality.

Second audit cycle

The second audit cycle demonstrated measurable improvement in documentation completeness following the educational and supervisory interventions. Documentation of anticipated blood loss improved to 10/35 (28.6%), while antibiotic prophylaxis and DVT prophylaxis were recorded in 7/35 (20%) and 5/35 (14.3%) of operation notes, respectively.

Substantial improvements were observed in descriptive operative elements, including documentation of the surgical approach in 28/35 (80%) (up from 20/50 (40%)), operative findings in 26/35 (74.3%) (up from 17/50 (34%)), and details of tissue altered in 25/35 (71.4%) (up from 16/50 (32%)). High levels of compliance were achieved for core procedural elements, such as documentation of the name of the operating surgeon in 35/35 (100%), the name of the operation in 34/35 (97.1%), and postoperative instructions in 33/35 (94.3%).

Despite these improvements, documentation of elective versus emergency status remained relatively low, being recorded in 11/35 (31.4%) of operation notes. Although surgeon signatures improved compared with the first cycle, full compliance was not achieved, with signatures present in 30/35 (85.7%) of operation notes.

Table 1 depicts the compliance with orthopaedic operation note documentation criteria across two audit cycles.

**Table 1:** Compliance with orthopaedic operation note documentation criteria across two audit cycles (18 core audit criteria) Values are presented as number/total (percentage). Absolute change represents the difference in percentage compliance between the first and second audit cycles. Postoperative instructions and surgeon signature were recorded and reported separately and were not counted as core audit criteria.

Documentation criterion	First cycle (n=50)	Second cycle (n=35)	Absolute change
Date of operation	47/50 (94%)	34/35 (97.1%)	+3.1%
Time of operation	37/50 (74%)	28/35 (80%)	+6%
Elective/emergency status	11/50 (22%)	11/35 (31.4%)	+9.4%
Name of operating surgeon	49/50 (98%)	35/35 (100%)	+2%
Name of assistant	42/50 (84%)	33/35 (94.3%)	+10.3%
Name of anesthetist	41/50 (82%)	32/35 (91.4%)	+9.4%
Name of operation	48/50 (96%)	34/35 (97.1%)	+1.1%
Details of skin incision	31/50 (62%)	26/35 (74.3%)	+12.3%
Surgical approach	20/50 (40%)	28/35 (80%)	+40%
Operative diagnosis	35/50 (70%)	29/35 (82.9%)	+12.9%
Operative findings	17/50 (34%)	26/35 (74.3%)	+40.3%
Mention of complications	33/50 (66%)	30/35 (85.7%)	+19.7%
Details of tissue removed/altered	16/50 (32%)	25/35 (71.4%)	+39.4%
Prosthesis details (if applicable)	39/50 (78%)	32/35 (91.7%)	+13.7%
Details of closure technique	28/50 (56%)	27/35 (77.1%)	+21.1%
Anticipated blood loss	0/50 (0%)	10/35 (28.6%)	+28.6%
Antibiotic prophylaxis	0/50 (0%)	7/35 (20%)	+20%
Deep vein thrombosis prophylaxis	0/50 (0%)	5/35 (14.3%)	+14.3%
Postoperative instructions	44/50 (88%)	33/35 (94.3%)	+6.3%
Surgeon signature	39/50 (78%)	30/35 (85.7%)	+7.7%

## Discussion

This two-cycle clinical audit demonstrates that targeted, low-cost quality improvement interventions can lead to meaningful improvements in the completeness of orthopaedic operation note documentation. It is important to emphasize that the observed improvements reflect enhancements in documentation practices rather than direct confirmation of the delivery of perioperative care itself. Following the implementation of focused educational sessions, dissemination of documentation standards, and senior-led reinforcement, substantial gains were observed across multiple documentation domains, particularly those related to operative description and procedural clarity. These findings are consistent with previous audit-based studies showing that structured feedback and education can significantly improve the quality of surgical documentation [5-7].

Despite overall improvement, documentation of key patient safety parameters, including anticipated blood loss, antibiotic prophylaxis, and DVT prophylaxis, remained suboptimal in the second audit cycle. Although absolute compliance for these parameters remained below 30%, the transition from complete absence of documentation at baseline to consistent recording represents a clinically meaningful relative improvement. Such changes indicate increased awareness of safety-critical elements and constitute an important initial step toward safer perioperative practice. Similar patterns of persistent underdocumentation despite educational interventions have been reported in other audits [6,7].

Improved documentation quality has direct implications for patient care. More complete operation notes support safer postoperative decision-making, improve the quality of clinical handover, and reduce ambiguity regarding perioperative management plans. Explicit documentation of operative findings, tissue altered, and prophylactic measures enhances continuity of care, facilitates risk stratification, and strengthens medico-legal defensibility, particularly in high-volume orthopaedic and trauma services.

The most notable improvements were observed in the documentation of the surgical approach, operative findings, and details of tissue altered, each showing an absolute increase of approximately 40%. Similar improvements have been reported across multiple surgical specialties, where audit-and-feedback interventions effectively improved compliance with operative documentation standards [5,8]. Evidence from studies evaluating electronic operative note proformas further supports the role of structured documentation approaches in reinforcing these gains [8,9]. This suggests that descriptive operative elements largely dependent on individual surgeon practice are particularly responsive to awareness-based quality improvement strategies.

Several system-level and contextual factors likely influenced documentation practices in this setting. High workload, time pressure in emergency and trauma theatres, reliance on handwritten notes, absence of electronic documentation systems, and prevailing documentation culture may limit the effectiveness of education-based interventions alone. These constraints are common in low-resource healthcare environments and should be considered when interpreting audit outcomes and planning sustainable improvements.

The key lesson of this audit is that while educational and supervisory interventions can achieve meaningful short-term improvements in documentation quality, sustained and comprehensive improvement requires system-level solutions. Among the recommended interventions, the introduction of a standardized, mandatory operation note template incorporating safety-critical prompts should be prioritized for immediate implementation, supported by ongoing audit cycles and focused staff engagement.

Limitations

This audit has several limitations. It was conducted at a single center, which may limit the generalizability of the findings. The sample size of the second audit cycle was smaller than that of the first. Additionally, the observed improvements may have been influenced by the Hawthorne effect, with clinicians modifying their documentation behavior due to awareness of being audited. Nevertheless, the consistent direction and magnitude of improvement across multiple documentation domains support the validity and practical relevance of the findings.

Recommendations 

To consolidate the observed improvements and address persistent documentation gaps, several measures are recommended. The implementation of a standardized operation note template with mandatory fields, particularly for anticipated blood loss and perioperative prophylaxis, may help reduce omissions and promote consistency. Adopting a checklist-based approach could further reinforce the documentation of antibiotic and DVT prophylaxis. In addition, regular, brief educational refreshers focused on patient safety and the medico-legal importance of complete documentation should be incorporated into departmental practice. Ensuring surgeon signatures on all operation notes is essential to maintain accountability and adherence to professional standards. Finally, periodic re-auditing, including a planned third audit cycle following template implementation, is recommended to assess the sustainability of improvements over time.

## Conclusions

This two-cycle clinical audit demonstrated that targeted educational and supervisory interventions can improve the completeness of orthopaedic operation note documentation. Meaningful gains were observed across several key documentation domains, particularly operative descriptions and procedural details. However, documentation of critical patient safety parameters, including anticipated blood loss, antibiotic prophylaxis, and DVT prophylaxis, remained suboptimal despite improvement from baseline.

These findings indicate that while awareness-based interventions are effective in improving documentation practices, they are insufficient on their own to ensure consistent recording of safety-critical elements. Importantly, the lessons from this audit are transferable beyond a single institution and are relevant to other orthopaedic units and surgical specialties, particularly in resource-limited settings where reliance on handwritten documentation and high clinical workload are common.

Embedding documentation standards into routine clinical practice through system-level solutions such as standardized, mandatory operation note templates has the potential to directly enhance patient safety by improving postoperative decision-making, continuity of care, and risk reduction. As a next step, the implementation of a structured operation note template followed by repeat audit cycles is recommended to evaluate sustainability and long-term impact. In practice, institutions should prioritize system-level documentation tools alongside education to achieve durable improvements in surgical safety and care quality.
